# Age, Health and Attractiveness Perception of Virtual (Rendered) Human Hair

**DOI:** 10.3389/fpsyg.2016.01893

**Published:** 2016-12-22

**Authors:** Bernhard Fink, Carla Hufschmidt, Thomas Hirn, Susanne Will, Graham McKelvey, John Lankhof

**Affiliations:** ^1^Faculty of Biology and Psychology, Institute of Psychology, University of GöttingenGöttingen, Germany; ^2^Wella Professionals R&D, Procter & Gamble Service Germany GmbHSchwalbach am Taunus, Germany; ^3^HFC Prestige Service Germany GmbHDarmstadt, Germany

**Keywords:** human, hair, modeling, perception, attractiveness, age, health

## Abstract

The social significance of physical appearance and beauty has been documented in many studies. It is known that even subtle manipulations of facial morphology and skin condition can alter people’s perception of a person’s age, health and attractiveness. While the variation in facial morphology and skin condition cues has been studied quite extensively, comparably little is known on the effect of hair on social perception. This has been partly caused by the technical difficulty of creating appropriate stimuli for investigations of people’s response to systematic variation of certain hair characteristics, such as color and style, while keeping other features constant. Here, we present a modeling approach to the investigation of human hair perception using computer-generated, virtual (rendered) human hair. In three experiments, we manipulated hair diameter (Experiment 1), hair density (Experiment 2), and hair style (Experiment 3) of human (female) head hair and studied perceptions of age, health and attractiveness. Our results show that even subtle changes in these features have an impact on hair perception. We discuss our findings with reference to previous studies on condition-dependent quality cues in women that influence human social perception, thereby suggesting that hair is a salient feature of human physical appearance, which contributes to the perception of beauty.

## Introduction

Even subtle manipulations of facial shape and skin condition can alter people’s perception of a person’s age, health, and attractiveness and such perception has consequences on social attributions (for a review, see Rhodes, 2006; Samson et al., 2010; Little et al., 2011). While the variation in facial shape and skin condition cues has been studied quite extensively, comparably little is known on the effect of hair on social perception. Face research typically controls for the effect of hair, assuming that hair color and/or style may affect people’s face assessments and therefore bias judgements in a way which is difficult to control for. With the aid of modern software, it is relatively simple to remove (or cover) hair in digital images and present face shape, feature placement and skin quality to panelists, asking them to evaluate age, health, and attractiveness. However, this approach disregards the effect of hair and thus captures only part of the natural variation in human physical appearance. Thus, there is little experimental evidence on role of head hair in social perception, which is possibly also caused by the technical difficulties of systematic feature variation in creating suitable stimuli.

The few available studies on social perception of hair have concentrated almost exclusively on hair color preferences. Rich and Cash (1993) investigated whether blonde women are over-represented in print media sampled over a period of some four decades. It was found that the percentage of blondes in magazines (e.g., Ladies’ Home Journal, Vogue, and Playboy) was higher than the incidence of blondes in a White female sample, suggesting that blonde is, somehow, an ‘ideal’ of feminine beauty (although there were some temporal shifts that could not entirely be explained by these data). Frost (2006) postulated the existence of a ‘rare-color advantage’ with regard to female hair. According to this view, those with a hair color of lower incidence in a given population should be perceived as more attractive than those with a hair color with greater incidence (but see Janif et al., 2015). Moreover, Sorokowski (2008) showed that men judged images of women with (digitally-enhanced) blonde hair significantly younger than the same images with brown hair (particularly in women around the age of 30). This result is, however, in contrast to the findings of Swami and Furnham (2007), who reported that blondes were rated as less physically attractive and more promiscuous than brunettes, a result that was replicated in two follow-up studies by the same authors (Swami et al., 2008a,b).

With regard to hair condition, Etcoff (1999) proposed that hair which is healthy, shiny and strong signals overall physical health and, conversely, that hair which lacks these signals may be perceived to have been damaged through illness (see also Symons, 1995). Hinsz et al. (2001) argued that female hair might well signal reproductive potential, because men are attracted to women who are physically young, healthy and attractive and that well-groomed, ‘good-looking’ hair may signal these parameters. These authors hypothesized that age-related characteristics of female hair will cause a woman to be perceived as less attractive because of the direct link between age and reproductive potential and found some support for their assertion, as data revealed a negative correlation between hair length and age. In addition, hair quality was correlated with female health, leading the authors to conclude that hair length and quality both signal aspects of female reproductive potential.

Mesko and Bereczkei (2004) had six conditions of hair in their stimuli when presenting facial photographs of young women to panelists, i.e., short, medium, long, disheveled, knot/bun, and unkempt. These styles were applied using standard hair of a commercially available ‘beauty software’ (Cosmopolitan, My Style). An inspection of this software reveals that it provides a rough application of hair templates to individual faces that can be loaded into the program. Thus, it does not capture the optical properties of hair due to the (three-dimensional, 3D) specular reflection patterns produced by hair interacting with light (e.g., Keis et al., 2004; McMullen and Jachowicz, 2004). Moreover, Mesko and Bereczkei (2004) did not include hair color as a variable in their study, hence the conclusion of this study remains primarily with regard to hair length only. The authors themselves mention that the available hairstyles were one of the limitations of their study.

With regard to people’s visual sensitivity to female hair color and condition, Fink et al. (2013) provide preliminary evidence for an effect on social perception by combining eye-tracking methodology (to study people’s visual attention) and rating data (to confirm people’s perception). The eye-gaze of men and women was tracked whilst they viewed randomized pairs of images of natural and colored hair tresses, each pair displaying the same tress before and after controlled cuticle damage. It was found that undamaged versions of natural and colored hair were perceived as significantly younger, healthier, and more attractive than corresponding damaged versions. Visual attention to images of undamaged colored hair was significantly higher than to their damaged counterparts, while in natural hair the opposite pattern was found. Although this finding highlighted the divergence in visual attention to undamaged colored female hair and damaged natural female hair and associated differences in social perception, the applicability of results to full head hair remained speculative.

One limitation of previous studies investigating effects of hair characteristics on perception has been the difficulty with creating suitable stimuli by systematically manipulating certain hair features (such as hair color or style) while keeping other properties constant. Hair creates complex visual patterns that result from anisotropic light scattering of hair fibers. Changing hair color in (two-dimensional) digital images with commercial software does usually not account for this effect, nor is it possible to account for possible alteration in visual appearance of hair due to style changes. In the present study, we employed a computer graphics approach to investigate peoples’ age, health and attractiveness perceptions to systematic variations of hair diameter (Experiment 1), hair density (Experiment 2), and hair style (Experiment 3), including possible interaction effects of these features with hair color. The creation of virtual (rendered) hair provides the opportunity to manipulate specific hair characteristics while retaining information of other feature and by maintaining color consistency.

The nature of the study was largely exploratory, as similar approaches to perception of virtual (rendered) hair have not been reported. Yet we expected to identify effects of systematic manipulations of hair features (diameter, density, and style) on perception. Reporting such effects was the primary aim of the study. Thus, we did not aim to elaborate more sophisticated hypothesis about the possible consequences in evolutionary terms (this may be premature) but to employ state-of-the art technology in the investigation of specific hair characteristics on person perception that can (and has to be) extended in future research.

### General Methodology

#### Stimuli

**FIGURE 1:**
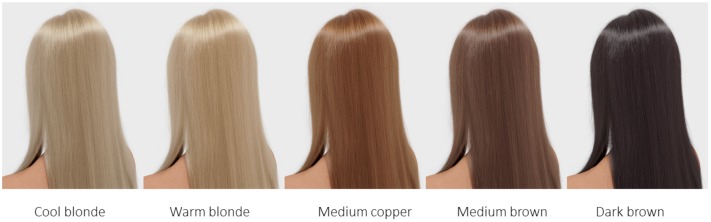
**Five hair shades of virtual (rendered) hair as used in all three experiments**.

Five shades of hair color were selected from the Wella Professionals portfolio, cool blonde, warm blonde, medium copper, medium brown, and dark brown (**Figure 1**), representing the most prevalent hair shades in Western Europe (see Frost, 2006). These shades were applied to all systematic variations of hair features in Experiments 1–3. Moreover, in Experiments 1 and 2, two hair types (straight and wavy) were introduced in addition to hair color and specific feature variations (i.e., hair diameter and hair density). In Experiment 3, we focus on effects of hair style variation in relation to hair color.

Virtual (rendered) full head hair stimuli varying in target features were created by employing light transport theory for modeling hair fibers (see Zinke and Weber, 2007; Zinke et al., 2009). In brief, this approach uses a hair mesh to generate realistic hair styles, thus providing the opportunity to manipulate the geometric and optical properties of hair fibers (100,000 in our base model). Monte Carlo path tracing technology (Kajiya, 1986) was used in a proprietary render engine to account for ‘color bleeding’ (i.e., color transfer caused by reflection of indirect light) and the Bidirectional Curve Scattering Distribution Function (BCSDF; Zinke and Weber, 2007; Zinke et al., 2009) for modeling radiance transfer in hair fibers. Definitions of optical properties for rendering hair stimuli were obtained from measurements of real hair. The final hair models were rendered in posterior view against a uniform gray background (R:G:B 200:200:200) with virtual D65 light (R:G:B 255:255:255) as global illumination and the same anatomical model (and skin texture) was used for the creation of all models. The render quality was set to 512 samples per pixel. The final images for presentation were 768 pixels × 1024 pixels in size and saved in bitmap file format.

#### Rating Studies

In all three experiments, participants viewed images of rendered hair on 27″ color-calibrated LCD monitors (Eizo Color Edge CG277, Eizo Corporation, Hakusan, Japan) with the monitor hood attached, set to a resolution of 2560 pixels × 1440 pixels (Gamma = 2.2, Color temperature = 6500 K; dE = 1.87). Monitors were connected to 15.6″’ laptop computers, running at 32-bit color depth (“true color”). The images were presented serially (one after the other) and in blocks (of attributes). Within each block, the order of appearance was randomized across participants. Participants were asked to guess the age of a person who has this hair, and to judge health and attractiveness of hair. Age judgements were provided in years. Health and attractiveness judgements were made on a 10-point Likert-type scale, presented next to the image (1 = low on attribute, 10 = high on attribute). Participants were not explicitly told that they were viewing virtual (rendered) hair. Experimental setups were realized using Medialab v2014 software (Empirisoft Corporation, New York, NY, USA). All experiments were conducted in the same location (a windowless room of approximately 3 m × 3 m) with a constant ambient light condition for all participants. The study protocol followed the principles for research involving human subjects as stated in the Declaration of Helsinki and was approved by the local ethical committee (application #155). Participants gave consent and received a payment of 5 Euros for their participation.

## Experiment 1

In this experiment, we investigated peoples’ age, health, and attractiveness perception of virtual hair varying in hair fiber diameter by applying three levels to our hair models. Hair diameter changes across the lifespan and varies between-individuals (Messenger, 2011; Mistry et al., 2012; Robbins, 2012). However, the evidence for a direct relationship of age and hair fiber diameter is mixed with regard to the type and direction of the relationship. In a sample of some 18,000 Japanese women (ages 10–60 years), hair diameter was found to be largest around the age of 40, and lower diameters were found before and after that age (Otsuka and Nemoto, 1988). Likewise, Robbins et al. (2012) reported that in a sample of 1,099 Caucasian women (ages 18–66 years), hair diameter was largest in women aged 43–46 years. These authors conclude that a lower rate of hair diameter increase, combined with the decrease in density, begins to significantly impact the perception of hair amount so that thinning becomes increasingly more noticeable in the mid 40ies to the mid to late 50ies. This possibly indicates that the menopause (and associated hormonal changes) is a critical event in a woman’s lifespan with regard to peak hair diameter (Robbins et al., 2012). Thus, hair ‘thickness’ may provide information about a woman’s age and people may be selective in their health and attractiveness assessments, such that they judge thinner hair as younger and more positively, especially when hair density reduction is an issue.

### Methods

#### Stimuli

**FIGURE 2:**
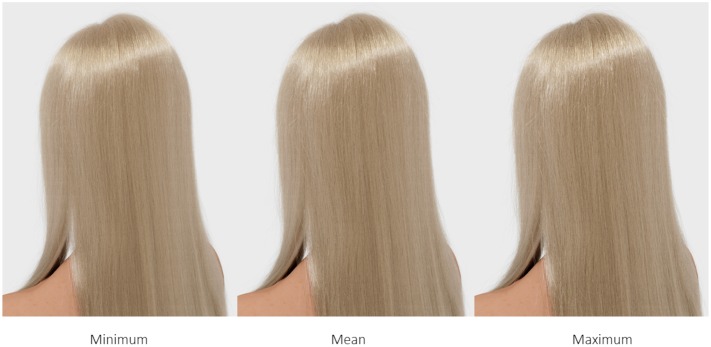
**Sample image of virtual (rendered) straight hair (cool blonde) varying in hair diameter (minimum, mean, and maximum diameter as measured in Caucasian hair)**.

We created a total set of individual 30 hair models (three diameter levels applied to five colors in two types; 100,000 fibers) and rendered them into digital images for presentation (**Figure 2**). We chose three diameter levels, i.e., 50 μm (minimum), 70 μm (mean), and 90 μm (maximum) hair fiber thickness, following the typical range of diameter reported for Caucasian hair (Robbins, 2012).

#### Participants

Our sample was 75 women, aged 17–29 years (*M* = 23.0, *SD* = 2.6), recruited mainly from the local student population at the University of Göttingen, Germany. Subgroups of 25 women independently rated the images for age, health, and attractiveness, respectively, to avoid carry-over effects.

### Results and Conclusion

**FIGURE 3:**
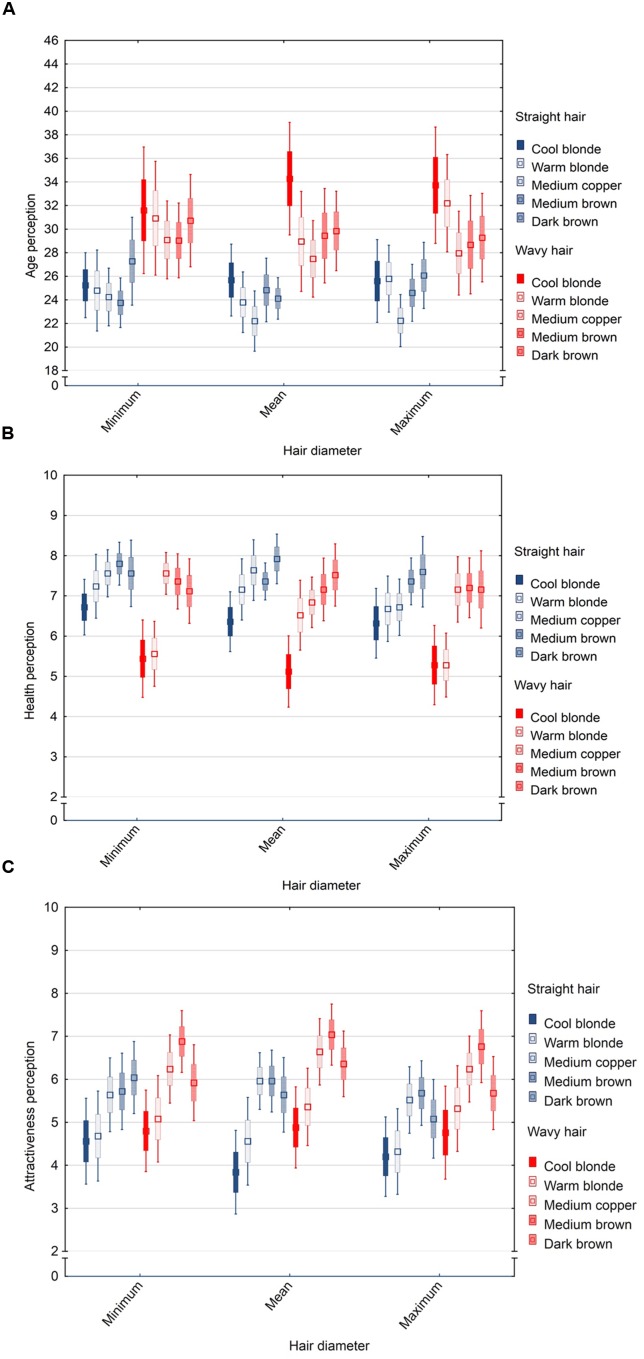
**Box-whisker-plot (M ± SE and CI) of age **(A)**, health **(B)**, and attractiveness **(C)** perceptions of three hair diameter levels varying color and hair type**.

A 3 (diameter) × 2 (type) × 5 (color) repeated measures ANOVA was performed, with the attributes as dependent variables and the hair features as within-subject factors to test for main and interaction effects. There were main effects of style [*F*(1,24) = 26.16, *p* < 0.001, η^2^ = 0.52] and color [*F*(4,96) = 3.35, *p* < 0.01, η^2^ = 0.13], but not diameter [*F*(2,48) = 0.66, *p* = 0.52, η^2^ = 0.03] on age assessments. Straight hair was perceived some 5 years younger (*p* < 0.001) compared to wavy hair. Medium copper hair was perceived as youngest and cool blonde hair as oldest with a difference of some 4 years in age perception (*p* < 0.05). No two-way or three-way interaction effects were detected (all *F* < 1.38, all *p* > 0.21) (**Figure 3A**).

With regard to health judgments, there were main effects of diameter [*F*(2,48) = 3.47, *p* < 0.05, η^2^ = 0.13], type [*F*(1,24) = 5.93, *p* < 0.05, η^2^ = 0.20], and color [*F*(4,96) = 18.62, *p* < 0.001, η^2^ = 0.44]. Thick hair was perceived as less healthy than thin hair (although in this case the pairwise comparisons were n.s.). Straight hair was perceived to be healthier than wavy hair (*p* < 0.05). Hair color showed the strongest effect on health perceptions with medium copper and brown hair being perceived as healthier than blonde hair (blonde shades vs. others, both *p* < 0.05; medium copper vs. brown shades, both *p* n.s.). Hair type and color revealed an interaction effect on health judgements [*F*(4,96) = 4.08, *p* < 0.01, η^2^ = 0.15] with blonde shades showing larger differences in health perception between straight and wavy type than other colors, both (*p* < 0.05), while other interactions were found to be non-significant (all *F* < 1.96, all *p* > 0.05) (**Figure 3B**).

For attractiveness judgements, we obtained main effects of diameter [*F*(2,48) = 4.61, *p* < 0.05, η^2^ = 0.16], style [*F*(1,24) = 5.35, *p* < 0.05, η^2^ = 0.18], and color [*F*(4,96) = 10.46, *p* < 0.001, η^2^ = 0.30]. Hair diameter and type both had a small effect on attractiveness perception compared with the larger effect of color. Thick hair was perceived least attractive, with no statistical difference of minimum vs. mean diameter (mean vs. maximum diameter *p* < 0.01). Brown and copper shades were perceived higher on attractiveness than blonde shades (medium brown and medium copper vs. blonde, all *p* < 0.05; dark brown vs. cool blonde *p* < 0.05). Interestingly, wavy hair was judged more attractive than straight hair (*p* < 0.05). An interaction effect was found for diameter and type [*F*(2,48) = 3.59, *p* < 0.05, η^2^ = 0.13], but not for other feature interactions (all *F* < 1.17, all *p* > 0.10) (**Figure 3C**). In straight type, thin hair was judged most attractive, whereas in wavy type, hair with mean diameter received the highest attractiveness judgments.

In conclusion, there was considerable variation in age, health and attractiveness perception of hair with regard to effects of hair diameter, type, and color. However, compared with other effects, the impact of hair diameter on perceptions was relatively minor and even absent for age judgements. Hair type (straight vs. wavy) seems to be the dominant factor in the estimating a person’s age from visual appearance of hair. Hair color had the strongest effect on perceptions of health and attractiveness. Compared to these effects, the manipulation of hair diameter resulted in rather subtle – yet statistically detectable – perceptual changes of health and attractiveness (but not age), with thick hair being perceived least positive. However, given the considerable variation in people’s assessments in relation to the small size of this effect, this result should be treated with caution.

## Experiment 2

In this experiment, we investigated peoples’ age, health and attractiveness perception of virtual hair varying in hair density by applying four density levels to our hair models. Hair density shows inter-individual variation due to natural and clinical condition (e.g., female pattern hair loss; Birch et al., 2002), and it decreases with age. Robbins et al. (2012) report that relative scalp coverage (as combination of density and diameter) is highest at age 35. However, hair density alone peaks in the late 20ies, before it begins to steadily decrease beyond age 30 years (Barman et al., 1965; Robbins et al., 2012). Whiting (2011) suggested that a threshold of 15% loss of hair density can be considered as a significant decrease, and this is typically found only later in life, whereas Marritt (1999) reported that 50% of scalp hair could be removed before the density reduction was noticeable. In this study, hair was plucked from a healthy subject, thus leaving hair diameter unchanged. This information is particularly applicable to the present experiment, as we were interested in people’s visual perception of hair density manipulations alone (i.e., by retaining other features), and investigate possible interaction effects with hair style and color. We hypothesized that hair density manipulations could show an effect with color and style by increasing the noticeability, and thus lead to less positive attributions.

### Methods

#### Stimuli

**FIGURE 4:**
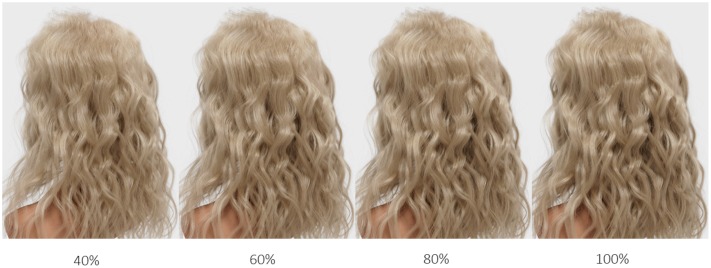
**Sample image of virtual (rendered) wavy hair (cool blonde) varying in hair density (40, 60, 80, and 100%)**.

We created a total set of individual 40 hair models (four density levels applied to five colors in two types) and rendered them into digital images for presentation (**Figure 4**). Original (i.e., 100%) hair density was set to 100,000 hair fibers – a typical number reported for Caucasian hair (Robbins, 2012) – and this density was reduced in 20% increments to obtain stimuli with 80, 60, and 40% of the original hair density.

#### Participants

Our sample was 90 women, aged 18 to 27 years (*M* = 21.2, *SD* = 2.0), recruited mainly from the local student population at the University of Göttingen, Germany. Subgroups of 30 women independently rated the images for age, health, and attractiveness, respectively, to avoid carry-over effects.

### Results and Conclusion

**FIGURE 5:**
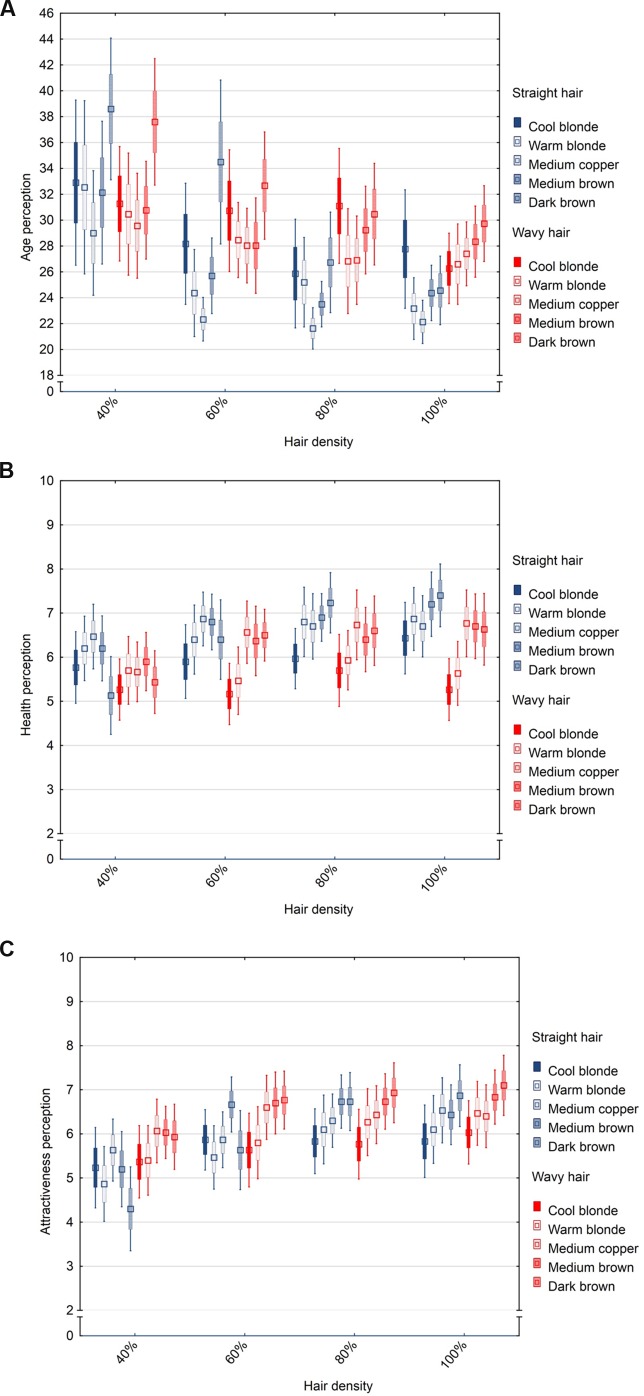
**Box-whisker-plot (M ± SE and CI) of age **(A)**, health **(B)**, and attractiveness **(C)** perceptions of four hair density levels varying color and hair type**.

A 4 (density) × 2 (type) × 5 (color) repeated measures ANOVA was performed, with the attributes as dependent variables and the hair features as within-subject factors to test for main and interaction effects. There were main effects of density [*F*(3,87) = 14.11, *p* < 0.001, η^2^ = 0.33], type [*F*(1,29) = 6.06, *p* < 0.05, η^2^ = 0.17], and color [*F*(4,116) = 7.88, *p* < 0.001, η^2^ = 0.21] on age assessments. Hair density showed the strongest effect on age perception, followed by that of color and type. The original (100%) density was considered as youngest, followed by 80, 60, and 40% of original density, with about 6 years of difference in perceived age between 40% and original density (all pairwise comparisons *p* < 0.05, except 80% vs. 100% n.s.). Wavy hair was judged older than straight hair (by about 2 years, *p* < 0.05). Medium copper hair was judged as youngest and dark brown as oldest (all pairwise comparisons *p* < 0.05, except for cool blonde hair n.s.; medium copper vs. brown shades *p* < 0.05). There were interaction effects of density and color [*F*(12,348) = 2.84, *p* < 0.001, η^2^ = 0.09] and density and type [*F*(3,87) = 6.81, *p* < 0.001, η^2^ = 0.19], but not for other interactions (all *F* < 1.74, all *p* > 0.06). In straight hair, the differences in age perception between density levels were more pronounced than in wavy hair (especially so for 60–100% density levels). Likewise, the range of variation in age perception was larger in dark brown hair compared with that in other shades (dark brown hair at 40% density was judged about 5 years older than other shades) (**Figure 5A**).

Main effects on health judgements were detected for density [*F*(3,87) = 4.95, *p* < 0.01, η^2^ = 0.15], type [*F*(1,29) = 6.23, *p* < 0.05, η^2^ = 0.17], and color [*F*(4,116) = 8.62, *p* < 0.001, η^2^ = 0.23]. Hair density manipulations had significant effects on health perceptions, following the pattern reported for age judgements, i.e., original (100%) density was judged to be healthiest and 40% density as least healthy (although pairwise comparisons were n.s.). Straight hair was rated as healthier compared to wavy hair (*p* < 0.05). Medium copper and brown hair were judged to be healthier than blonde shades, and especially cool blonde was perceived as least healthy (cool blonde vs. other shades, all *p* < 0.05). Density and color had an interaction effect on health perception [*F*(12,348) = 3.12, *p* < 0.001, η^2^ = 0.10], while other interactions were found to be non-significant (all *F* < 2.22, all *p* > 0.07). Dark brown hair showed the largest variation in health perception across density levels, with 40% being least healthy and 100% healthiest (of all shades) (**Figure 5B**).

We obtained main effects of density [*F*(2,48) = 25.81, *p* < 0.001, η^2^ = 0.47] and color [*F*(4,116) = 3.64, *p* < 0.01, η^2^ = 0.11], but not type [*F*(1,29) = 2.10, *p* = 0.16] on attractiveness assessments. Hair density had a strong effect on attractiveness perception following the pattern reported for age and health perceptions, i.e., original (100%) density was perceived as most attractive and 40% density as least attractive (40% vs. other density levels, all *p* < 0.001; 60% vs. 100%, *p* < 0.01). Wavy hair was not considered significantly different in attractiveness than straight hair (pairwise comparison n.s.). Blonde hair was judged less attractive than medium copper and brown hair, although pairwise comparisons were n.s.

There were interaction effects of density and color [*F*(12,348) = 3.94, *p* < 0.001, η^2^ = 0.12] and density and type [*F*(3,87) = 4.33, *p* < 0.01, η^2^ = 0.13] on attractiveness perceptions, but no effects from other interactions (all *F* < 1.82, all *p* > 0.10). The differences in attractiveness perceptions were more pronounced in straight hair than in wavy hair (especially so for 40–60% density) and particularly evident in medium and dark brown hair compared with other shades (**Figure 5C**).

In sum, hair density shows effects on age, health, and attractiveness perceptions. Straight is perceived more positively than wavy hair, but this does not necessarily apply to age perception. The original density, defined as 100% following reports from the literature on hair fiber counts in Caucasians, was judged to be youngest, healthiest and most attractive.

## Experiment 3

Hair style is highly variable, and systematic effects of visual perception are, therefore, difficult to investigate. Thus, there is little information on the effect of hair style on physical appearance in the literature. Mesko and Bereczkei’s (2004) study provides some evidence on the effect of hair length on attractiveness assessment (medium-length and long hair were preferred), leading the authors to the conclusion that hair style may display phenotypic quality. Changing hair style alters hair texture, and has consequences on the specular reflection patterns produced by hair interacting with light (e.g., Keis et al., 2004; McMullen and Jachowicz, 2004). Thus, straight and wavy hair of the same length, for example, can have different effects on visual perception due to differences in optical properties. The same applies to hair color. With blonde hair, specular reflection patterns (in the form of ‘shine’) are less visible than in darker hair. Mesko and Bereczkei’s (2004) did not include hair color as variable in their study but adjusted hair color to approximate the natural hair color of their subjects. While this approach kept hair color constant within-subjects, it does not provide information on possible interaction effects of hair style and hair color on assessments. In the present experiment, we included eight hairstyles women typically request in the salon and investigated possible interaction effects with hair color. Although these hair styles vary in length and texture, our focus was not on systematic effects of hair length (sensu Mesko and Bereczkei, 2004) but to determine the strength of effects of hair style (and color) on people’s perception, and compare them with those reported in Experiments 1 and 2. We expected that hair style would show a stronger effect on people’s judgements than that of hair color, and also those observed from hair fiber diameter and hair density.

### Methods

#### Stimuli

**FIGURE 6:**
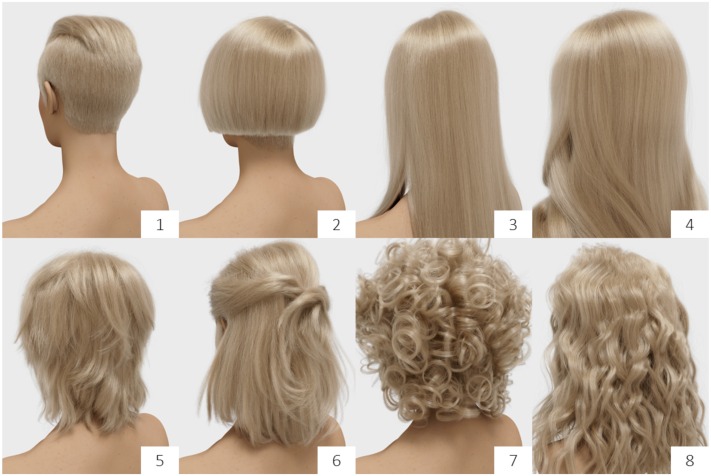
**Eight styles of virtual (rendered) hair (cool blonde), including the “straight” (3) and “wavy” (8) hair types as used in Experiments 1 and 2**.

We created a total set of individual 40 hair models (eight styles applied to five colors, 100,000 fibers) and rendered them into digital images for presentation (**Figure 6**). Hair styles did not follow specific categories in terms of, for example, hair length variations, but represented women’s typical requests in the salon.

#### Participants

Our sample was 90 women, aged 17 to 28 years (*M* = 21.5, *SD* = 2.6), recruited mainly from the local student population at the University of Göttingen, Germany. Subgroups of 30 women independently rated the images for age, health, and attractiveness, respectively, to avoid carry-over effects.

### Results and Conclusion

A 8 (style) × 5 (color) repeated measures ANOVA was performed, with the attributes as dependent variables and the hair features as within-subject factors to test for main and interaction effects. There were main effects of style [*F*(7,203) = 16.26, *p* < 0.001, η^2^ = 0.36] and color [*F*(4,116) = 4.97, *p* < 0.001, η^2^ = 0.15] on age assessments, main effects on health judgements for style [*F*(7,203) = 35.92, *p* < 0.001, η^2^ = 0.55] and color [*F*(4,116) = 7.38, *p* < 0.001, η^2^ = 0.20], and a significant effect main of style [*F*(7,203) = 35.66, *p* < 0.001, η^2^ = 0.55], but not for color [*F*(1,116) = 1.91, *p* = 0.11] on attractiveness judgements. Thus, hair style created a stronger effect than color, and this was found for age, health, and attractiveness perceptions of hair varying in these two features. The effect of style was strongest for health perception, followed by an equally sized effect on attractiveness and a smaller effect on age assessment. Styles 5 and 7 in particular were judged older than other styles (*p* < 0.01), Styles 5 and 6 as least healthy (five pairwise comparisons *p* < 0.01) and Styles 1 as least attractive (five pairwise comparisons *p* < 0.01). Color showed a significant effect on age and health judgements (but not so for attractiveness), albeit small in magnitude with little variation in mean age perception across shades (age: pairwise comparisons n.s., except cool blonde vs. warm blonde and medium brown vs. dark brown, *p* < 0.05; color: cool blonde vs. brown shades, both *p* < 0.05).

**FIGURE 7:**
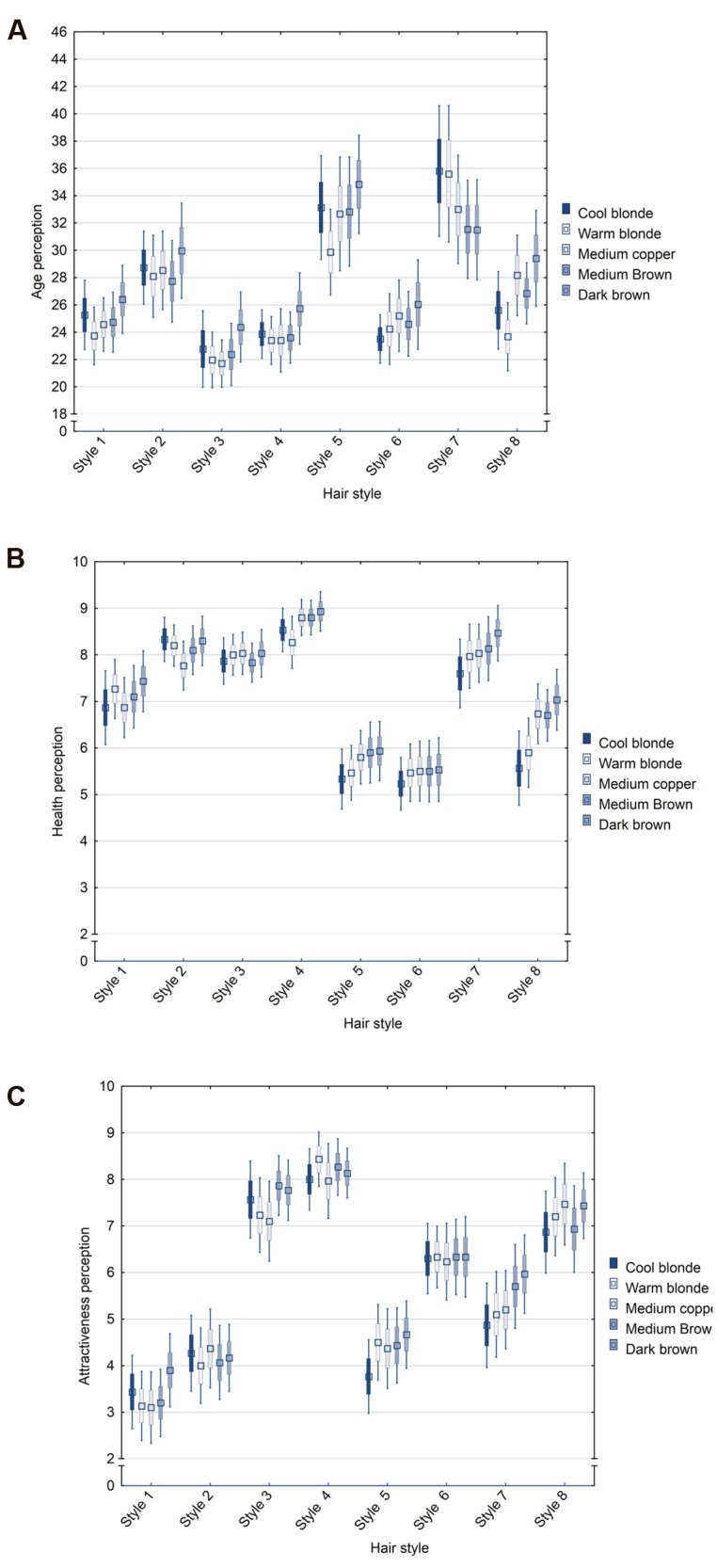
**Box-whisker-plot (M ± SE and CI) of age **(A)**, health **(B)**, and attractiveness **(C)** perceptions of eight hair styles varying in color**.

Interaction effects were detected for style and color for assessments of age [*F*(28,812) = 2.15, *p* < 0.001, η^2^ = 0.07], health [*F*(28,812) = 1.97, *p* < 0.01, η^2^ = 0.06], and attractiveness [*F*(28,812) = 1.82, *p* < 0.01, η^2^ = 0.06]. These effects showed that for some styles the effect on perception was more pronounced than for others, depending on hair color (this was especially so for warm blonde hair), although they were relatively minor compared to the main effects, particularly that of hair style (**Figures 7A–C**).

We conclude that hair style has a strong effect on people’s perception of age, health, and attractiveness of hair, and this effect is generally stronger than that of hair color. Our results also show that hair style effects are not necessarily independent from color (albeit that such interaction effects are small in magnitude).

## General Discussion

Systematic manipulations of hair diameter, hair density, and hair style revealed a series of main and interaction effects on perceptions of age, health, and attractiveness of hair. A general observation across experiments was that straight hair was perceived as younger, healthier, and more attractive than wavy hair and darker shades (medium copper and brown) were perceived more positively than blonde hair. Some previous studies have reported a (male) preference for blonde hair in women (e.g., Sorokowski, 2008), and blonde women were also found to be over-represented in magazines (Rich and Cash, 1993), leading to the speculation that blonde hair could be seen as an ‘ideal’ of beauty (but see Millward’s 2013 report on black and brown hair in female porn stars outnumbering blondes by 2:1). Our data on women’s perceptions of hair does not support this view given the less positive perception of cool and warm blonde hair, basically independent manipulations of other hair features. The preference for blonde hair may be evident only in men, as it has been proposed in the ‘rare-color advantage’ hypothesis (Frost, 2006). Thus, our data cannot support the view on either a presence or absence of such an effect (see for a lack of evidence, Janif et al., 2015). One could argue that women assessing other women’s hair tend to ‘derogate’ them by assigning less positive statements to features that are admired by men due to intra-sexual competition (as it has been shown for female facial attractiveness, Fisher, 2004). We consider also this effect unlikely to be responsible for the observed pattern in our data, given the subtlety of ovulatory-cycle-dependent shifts in female preferences and the necessity of within-subject experimental designs to detect them (see for a meta-analysis Gildersleeve et al., 2014). Other studies have reported blondes to be rated as less attractive and more sexually promiscuous (Swami and Barrett, 2011), including cross-cultural data on higher attractiveness of brown hair than blonde hair (Swami et al., 2008b). Our data on women’s perceptions of hair confirm these findings, as across experiments darker shades (medium copper and brown hair) were considered as healthier and more attractive than blonde hair, independent from hair thickness, density and style. However, it is noteworthy that out study was conducted in a specific (Western European) population. Whether the reported effects are detected in other populations remains to be tested in cross-cultural investigations.

With regard to hair thickness (Experiment 1), we found that effects of hair diameter on health and attractiveness judgements (but not age), with thick hair being perceived less positive. However, pairwise comparisons of hair diameter levels did not reveal a coherent pattern so that ‘thin’ hair is always perceived more positively. In fact, an interesting interaction effect was found for hair type and hair diameter for attractiveness perception, with thin straight hair being judged most attractive and mean diameter receiving highest attractiveness assessments in wavy hair. Thus, the possible effect on visual assessment of hair attractiveness with a person’s given hair thickness, may depend on the choice of hair style. Although our focus was on the effect of hair diameter on hair perception, it is possibly not surprising that by manipulating this feature systematic effects on visual perception are subtle (or even absent), as hair diameter together with hair density creates the visual impression of hair volume. Robbins et al. (2012) proposed a new metric (‘hair amount,’ as related to volume), for quantifying the combined impact of hair diameter and density on perception of hair. These authors showed that in women, the relative hair amount peaked between the ages of 25 and 45 years. Hair diameter was found to increase until the age of 45, whereas hair density peaked about 27 years of age. Thus, the age of maximum hair density is lower than that for diameter. The earlier decrease in hair density is possibly minimized by the increase in hair diameter up to the age at menopause, after which hair loss is more noticeable. Robbins et al. (2012) suggest that age-related hormonal changes affect specific facets of the hair follicle (see also Ohnemus et al., 2006; Piérard-Franchiomont and Piérard, 2013), and this has consequences on both hair diameter and density, which together affect visual appearance of hair, especially in peri-/post-menopausal women.

We created virtual (rendered) models of female head hair, and thus do not have chronological age information of our target stimuli. Considering people’s age assessments across experiments reveals that none of our models were clearly judged to be in the post-menopausal age range. In this regard, the virtual models of hair should be extended in future studies by including other age-related changes of hair, resulting, for example, from physical and chemical damage. Previous studies in natural and colored hair has reported an effect of physical damaging on visual attention and assessment of hair (Fink et al., 2013). Yet it is interesting that even in the present set of hair stimuli, hair density manipulations had a strong effect on age, health and attractiveness perceptions (Experiment 2), and this effect was stronger than that of color and type (straight vs. wavy). With reference to the Robbins et al.’s (2012) ‘new metric,’ combining hair diameter and density, we therefore suggest that the effect of hair density on visual perception is stronger than that of hair diameter in creating age-related visual appearance of hair volume. Moreover, we believe that health-related change of hair density is more severe in terms of people’s perception than inter-individual variation and age-related decrease of hair diameter. In other words, hair loss due to disease (or even stress; Sandok, 1964; Spencer and Callen, 1987) can basically occur at any time in life, while similar changes in hair diameter are usually not observed. Thus, variation in hair density may reveal more accurate information about an individual’s physical condition than hair diameter does.

The effects of density on age perception were more evident in straight than in wavy hair. So it seems that a decrease in density is less noticeable in textured hair. An interesting question in this context is whether a woman’s choice of hair style is condition-dependent (see Hinsz et al., 2001)? Moreover, do women (consciously or unconsciously) cover certain conditions by choosing hair styles, which may make it less likely to notice issues, or put differently, can hair style be used to fake (good) physical condition associated with hair quality? To our knowledge, such a hypothesis has not yet been tested. Mesko and Bereczkei (2004) suggest hairstyle as an adaptive means of displaying phenotypic quality. These authors did, however, focus on hair length, and although they included ‘disheveled’ and ‘unkempt’ hair styles in their study, these styles do not have the properties of healthy-looking wavy or curly hair, which would be important to consider (in addition to other styles) in addressing questions on the deceptive use of hairdo. In that vein, related hypotheses can be formulated with regard to the use of hair color. While our experiments indicate that brown and copper shades were generally perceived more positively than blonde shades, we also detected that dark brown hair showed the largest variation health perception across density levels. Hence, in dark shades changes of hair density may be more noticeable than in light shades, and especially light blonde hair may not as easily reveal changes in age- or condition-related hair properties. Our technical model can be used to test people’s noticeability and visual perception of systematic feature manipulations, but it has limitations when it comes to hypotheses on condition-dependent use and display of certain hair colors and styles. We still hope that this present and future work will stimulate investigations into ‘real’ subjects that will address these questions.

Finally, our data on effects of hair style diversity (Experiment 3) show a strong effect on visual perceptions of hair, especially for health and attractiveness assessments. Long hair was considered most attractive, and medium-length hair styles that may have appeared unkempt to observers were considered least healthy. We interpret these findings as quality cue to grooming and maintenance of hair, with long hair being especially difficult to display in good condition, and therefore considered most attractive if healthy-looking. Our primary interest with hair style effects on perception was, however, the magnitude of these effects rather than the effect of specific style categories on visual perception (creating such categories based on objective hair characteristics is a project in its own right). That said, hair style effects were stronger than those reported for other features. Previous studies showed that hair style can improve the perception of female facial attractiveness (Mesko and Bereczkei, 2004), especially in women who were judged less positive for facial attractiveness. Moreover, it has been reported that perception of physical attractiveness is ‘leaky’ (Saegusa et al., 2015), i.e., there is mutual influence of facial attractiveness and hair attractiveness. In other words, even though people do not consciously spend attention of hair, their visual appearance still has an effect on face perception (and vice versa). We believe that two conclusions can be drawn from such findings.

First, in real-life settings where hair is visible, it has an effect on social perception. Given people’s sensitivity in terms of age, health, and attractiveness to systematic variation of hair characteristics (as we show here), head hair contributes to overall perception of physical appearance. Studies on physical attractiveness have mostly considered face and body morphology and movement in the attempt to detect cues that provide information about mate quality. We believe that hair needs to be added to the list of quality cues given the reported effects on perception from this present and previous studies, even though the information about an individual’s quality derived from hair must not be as accurate as other physical cues. Second, head hair may have been disregarded in previous studies because of technical difficulties with investigating it, but also because it is easily modifiable and may therefore not be seen as ‘honest’ quality cue. However, our present data show that people are selective in their assessment of hair condition and judge certain feature more positively than others on key attributes in social perception. Furthermore, we hypothesize that physical appearance, including head hair, comprises a condition-dependent ornament of quality, as it has been proposed for female faces and bodies (Thornhill and Grammer, 1999). To date, there is little information on the potential signaling quality of hair, although studies on age- and health-dependent changes in visual appearance of hair suggest that hair quality may be estrogen-dependent, as it has been proposed for other physical features (e.g., Law Smith et al., 2006; Röder et al., 2013). If this were true, it would explain the relationship of certain hair characteristics with attractiveness and health perceptions, and possibly also age-related incidences of hairstyle choice in women.

Although the present data contribute to the understanding of how we perceive hair, a number of questions remain to be investigated in future research. For example, the possibility to animate virtual (rendered) hair by presenting them to participants in three-dimensional view and under different (standardized) light conditions facilitates the investigation of consistency in perceptions of hair specific properties and styles. In the present study, a virtual D65 light (‘daylight’) was used for illumination. This approach can be extended by introducing additional lights, thus creating a more naturalistic scenario for the assessment of hair models. In fact, the careful control of light conditions is one of the key elements of the present hair modeling approach, and although we make the assumption that standardized light conditions facilitated the study of people’s perceptions of systematic variations of certain hair characteristics, it remains to be investigated whether this holds true also for other light settings than that used in the present study. This will be particularly interesting in future investigations that combine faces and hair, thus trying to identify the relative impact of facial and hair information on people’s perceptions of physical appearance under different light conditions. The application of different light settings to our virtual (rendered) hair models may also provide additional realism to virtual hair. Feedback from lay panelists and professional hair stylists on the hair models used in the present study revealed that many of them believed that our stimuli showed photographs of ‘real’ women’s head hair. Although we make the implicit assumption that the creations of virtual hair models worked equally well for all systematic conditions, we cannot rule out the possibility that some feature combinations may appear more realistic than others. This needs to be tested in future research.

With regard to evolutionary implications on the role of hair quality in perception of female appearance, a replication of the present study with male judges is clearly needed. Because of their link with age, we do not necessarily expect gender differences in the assessment of variation in hair diameter and density, given men’s preferences for youthful and healthy looking female features, which was evident also in women’s assessments of hair. The choice of a certain hair style, however, may polarize among observers, and it will be interesting to see if the observed female preference for long (but not always blonde) hair is shared by men. Studies on hair color preferences have produced mixed results, and we hope that subjecting our hair models to cross-cultural investigation will help resolving the question on gender difference in hair color perception.

## Conclusion

We embrace the assertion that hair is a salient feature of human physical appearance, which contributes to the perception of beauty. Our present data show that women are remarkably sensitive to subtle variations of hair diameter and density, in addition to variation in hair color and style and judge them selectively on age, health, and attractiveness. These features depend on age-related hormonal changes, and may therefore signify reproductive potential. Hair style seems to have the strongest effect on visual perception. Whether women’s choice of certain hair styles is condition dependent and used to cover hair characteristics in order to enhance their overall physical appearance will be subject of future studies.

## Author Contributions

BF, CH, TH, SW, GM, and JL conceived the study. CH and TH prepared the stimuli. CH collected rating data. BF and CH analyzed the data. BF wrote the manuscript. TH, SW, GM, and TH revised the manuscript for intellectual content.

## Conflict of Interest Statement

The authors declare that the research was conducted in the absence of any commercial or financial relationships that could be construed as a potential conflict of interest.
